# MRI assessment of body composition for prediction of therapeutic response to biologic agents in patients with Crohn’s disease

**DOI:** 10.1186/s13244-025-01930-w

**Published:** 2025-03-19

**Authors:** Naomi S. Sakai, Andrew A. Plumb, Norin Ahmed, Kashfia Chowdhury, Yakup Kilic, Maira Hameed, Anisha Patel, Anisha Bhagwanani, Emma Helbren, Rachel Hyland, Gauraang Bhatnagar, Harbir Sidhu, Hannah Lambie, James M. Franklin, Maryam Mohsin, Elen Thomson, Darren Boone, Damian Tolan, Safi Rahman, Nik Ding, Gordon W. Moran, Stuart Bloom, Ailsa Hart, Alex Menys, Simon Travis, Steve Halligan, Stuart A. Taylor, On Behalf of the Motility trial investigators, Norin Ahmed, Kashfia Chowdhury, Yakup Kilic, Maira Hameed, Anisha Patel, Anisha Bhagwanani, Emma Helbren, Rachel Hyland, Gauraang Bhatnagar, Harbir Sidhu, Hannah Lambie, Maryam Mohsin, Elen Thomson, Darren Boone, Damian Tolan, Safi Rahman, Stuart Bloom, Ailsa Hart, Alex Menys, Simon Travis, Steve Halligan, Tariq Ahmad, Saiam Ahmed, Fardowsa Ahmed-Timms, Rachel Baldwin-Cleland, Uday Bannur Chikkeragowda, Nina Barratt, Teresita Beeston, Biljana Brezina, Amanda Cetroni, Junaid Choudhury, Bessie Cipriano, Maria Dilawershah, Heather Fitzke, Tracy Foster, James Franklin, Anmol Gangi-Burton, Nicola Gibbons, Edmund Godfrey, Arun Gupta, Anthony Higginson, Judith Holmes, Elizabeth Isaac, Ilan Jacobs, Roman Jastrub, Mayamol Joseph, Jaspreet Kaur, Klaartje Bel Kok, Felix Kpodo, Shankar Kumar, Sarah Langlands, Eric Loveday, Sara McCartney, Peter Mooney, Gordon Moran, Felicia Onoviran, Miles Parkes, Jaymin Patel, Kamal Patel, Kamini Patel, Nishant Patodi, Sue Philpott, Andrew Plumb, Richard Pollok, Robert Przemiosolo, Helen Rafferty, Javen Ramsami, Charlotte Robinson, Suzanne Roffe, Lindsay Rogers, Konstantina Rosiou, Naomi Sakai, Abi Seward, Stuart Taylor, Belinda Theis, Nora Thoua, Anvi Wadke, Lana Ward, Annamaria Wilce, Steven Williams

**Affiliations:** 1 Centre for Medical Imaging, University College London, Division of Medicine London, London, UK; 2 Department of Radiology, University College London Hospitals, London, UK; 3 Comprehensive Clinical Trials Unit, University College London, London, UK; 4 Radiology Department, Western General Hospital, Edinburgh, UK; 5 Radiology Department, Wexham Park Hospital, Frimley Health NHS Foundation Trust, Slough, UK; 6 Radiology Department, Hull University Teaching Hospital NHS Foundation Trust, Hull, UK; 7 St James’s University Hospital, Leeds, UK; 8 Frimley Park Hospital, Surrey, UK; 9 Institute of Medical Imaging and Visualisation, Bournemouth University, Bournemouth, UK; 10 Epsom and St Helier University Hospitals NHS Trust, Epsom, UK; 11 Gastroenterology Department, St Vincent’s Hospital, Melbourne, VIC Australia; 12 Translational Medical Sciences, School of Medicine, Faculty of Medicine and Health Sciences, University of Nottingham, Nottingham, UK; 13 NIHR Nottingham BRC, University of Nottingham and Nottingham University Hospitals, Nottingham, UK; 14 Department of Gastroenterology, University College London Hospitals NHS Foundation Trust, London, UK; 15 St Mark’s the National Bowel Hospital, London, UK; 16 Motilent, London, UK; 17 Kennedy Institute of Rheumatology, University of Oxford, Oxford, UK; 18 Royal Devon and Exeter NHS Foundation Trust, Exeter, UK; 19 Barts and The London NHS Trust, London, UK; 20 Nottingham University Hospitals NHS Trust, Nottingham, UK; 21 Portsmouth Hospitals NHS Trust, Portsmouth, UK; 22 Addenbrookes Hospital, Cambridge, UK; 23 Royal Berkshire NHS Foundation Trust, Reading, UK; 24 Homerton NHS Foundation Trust, London, UK; 25 North Bristol NHS Trust, Frenchay Hospital, Bristol, UK; 26 St George’s University Hospital NHS Foundation Trust, London, UK

**Keywords:** Magnetic resonance imaging, Crohn’s disease, Biological therapy, Body composition

## Abstract

**Objectives:**

Altered body fat and muscle mass in Crohn’s disease (CD) have been linked to adverse disease course and outcomes. Prediction of treatment response or remission (RoR) of small bowel CD (SBCD) to biologic therapy remains challenging. We aimed to establish the prognostic value of body composition parameters measured using MR enterography (MRE) for RoR at 1 year in patients with SBCD commencing biologic therapy.

**Methods:**

Participants were identified from those recruited to a prospective, multicentre study investigating the predictive ability of motility MRI for 1 year RoR in patients starting biologic therapy for active SBCD (MOTILITY trial). Myopenia, skeletal muscle:fat and visceral:subcutaneous fat were measured from baseline MRE. RoR at 1 year was judged using a composite of clinical and morphological MRE parameters. We compared the likelihood of RoR in patients with and without myopenia or low skeletal muscle:fat using logistic regression models.

**Results:**

Ninety-six participants were included (mean age 38.2 years; 40 (42%) female). There were 34 (35%) responders. There was no significant difference in RoR at 1 year between those patients with and without skeletal muscle myopenia (OR: 0.85, 95% CI: 0.27, 2.66, *p*-value: 0.78), or those with or without low skeletal muscle:fat (OR: 0.71, 95% CI: 0.19, 2.71, *p*-value: 0.62).

**Conclusions:**

Body composition parameters demonstrated no value for predicting therapeutic RoR in patients commencing biologic therapy for SBCD.

**Critical relevance statement:**

Prediction of response to biologic therapy in small bowel Crohn’s disease (SBCD) remains challenging. Body composition parameters cannot predict biologic therapeutic response or remission for SBCD reliably.

**Key Points:**

- Altered body fat and muscle mass in Crohn’s disease have been linked to adverse outcomes.
- Prediction of response to biologic therapy in small bowel CD (SBCD) would be useful for treatment optimisation.
- Body composition parameters measured using MRI cannot reliably predict biological therapeutic response or remission for SBCD.

**Graphical Abstract:**

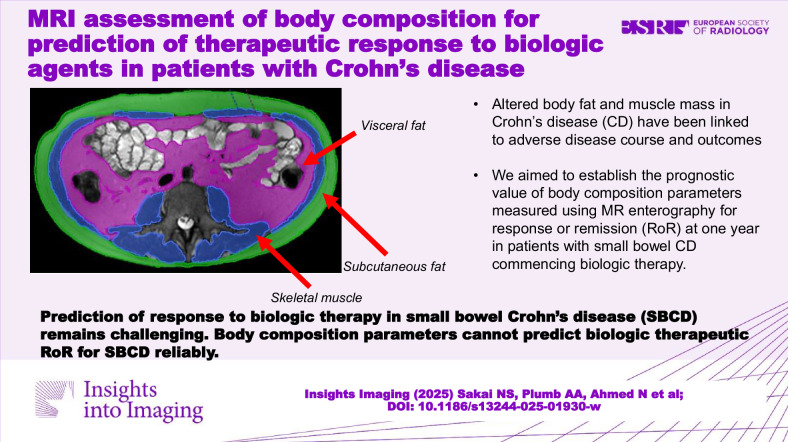

## Introduction

Patients with inflammatory bowel disease (IBD) have altered quantities of fat and muscle throughout their body compared to unaffected individuals [1, 2]. This is multifactorial and may be due to malnutrition, catabolic status and/or malabsorption, all of which alter body composition [3, 4]. Decreased muscle mass (myopenia) can be demonstrated in many patients with small bowel Crohn’s disease (SBCD), even those in clinical remission [5]. In patients with CD who require surgery, altered body composition is associated with increased postoperative complications [6, 7].

Malnutrition, defined as a body mass index (BMI) < 18.5 kg/m^2^, affects approximately 65–75% of patients with CD at some point [8], but this simple clinical measure may be insensitive to body composition. For example, a study of patients with myopenia found 49% had normal BMI [9], and some were even defined as overweight or obese [10]. Additionally, although more than half of patients with CD have normal or low BMI, visceral fat is significantly higher than healthy controls [11]. Whilst obesity measured using BMI has been suggested to be associated with worse clinical outcomes including increased rates of hospitalisation [12] and surgery [13], increased visceral fat may be more important (especially as this can be increased even if BMI is normal) and has been shown to be associated with increased complications and worse quality of life in CD [14].

Biological drugs, including antibodies directed against Tumour Necrosis Factor alpha (anti-TNFα), IL-12/IL-23 inhibitors and anti-integrin therapies, have transformed SBCD treatment [15]. Whilst biologics are available widely, safe and highly effective, current first-line therapies do not work for all patients [16]. Early identification of patients unlikely to achieve sustained response or remission (RoR) would be highly beneficial to optimise treatment allocation early, instigating alternative therapeutic approaches. However, it is currently not possible to identify which individuals will achieve RoR, using either clinical factors or imaging findings  [17].

Body composition affects the volume of distribution of many medications, and it is therefore plausible that this contributes to the pharmacokinetic failure of anti-TNFα therapy due to inadequate dosing [18]. Initial work from two single-site retrospective cohorts found that (1) myopenia is associated with nonresponse to anti-TNFα therapy [19] and (2) patients with myopenia experienced earlier treatment failure [20].

Magnetic resonance enterography (MRE) is widely used for diagnosis and follow-up of SBCD. Sequences acquired during conventional MRE can assess body composition, including myopenia, and therefore may potentially predict which patients are more likely to achieve longer-term RoR.

We investigated whether body composition of patients with SBCD could predict response to biologic therapy. We hypothesised that myopenia or low muscle:fat prior to therapy initiation is associated with failure to reach RoR at 1 year.

## Methods

This was a prespecified substudy of the MOTILITY trial (ISRCTN14481560), a prospective multicentre (13 UK hospitals), non-randomised, cohort study of patients aged 16 years or older with active, non-stricturing SBCD, commencing anti-TNFα or anti-interleukin therapy. The primary outcome was the ability of changes in cine motility MRI (mMRI) between baseline and post-induction to predict 1-year response or remission. The study was ethically approved (NHS West Midlands Research Ethics Committee: 17/WM/0106) and the protocol is publicly available (https://www.isrctn.com/editorial/retrieveFile/18aadd81-26ad-48d6-ab3e-6d90eb5b2d06/33110). All patients gave written informed consent.

### Patients

Patients commencing anti-TNFα or anti-interleukin (IL) 12/23 therapy for active SBCD, documented by imaging or endoscopy within 90 days of recruitment, underwent MRE at baseline and post-induction (12–30 weeks) and again at around 1 year. RoR at around 1 year (44–78 weeks) was judged based on a combination of clinical, ileocolonoscopic (if available) and MRE morphological parameters as described below.

Inclusion criteria for the main study were: (1) age ≥ 16 years with active luminal SBCD; (2) disease distribution and activity documented by ileocolonoscopy, MRE, intestinal ultrasound, computed tomography (CT), barium fluoroscopic follow-through, or video capsule endoscopy performed as part of usual clinical care within the previous 90 days prior to starting eligible biological therapy, or within 14 days after first treatment dose; (3) commenced or scheduled to commence or recommence eligible biological treatment (including biosimilars) with anti-TNFα (e.g., infliximab or adalimumab) or anti-IL 12/23 therapy (Ustekinumab); and (4) the primary target of therapy was small bowel disease.

Exclusion criteria for the main study were: (1) biological therapies other than anti-TNFα and anti-IL 12/23 therapy; (2) primary target of therapy was isolated colonic or perianal fistulating disease; (3) contraindication to MRI; (4) inability to give informed consent; (5) small bowel surgery within the preceding 90 days; or (6) small bowel stricture causing upstream dilatation on imaging or endoscopy (defined as a > 50% increase in diameter in comparison to the adjacent small bowel segment).

All participants recruited to the main study were potentially eligible for this substudy; however, patients were excluded if either RoR at 1 year could not be assigned (e.g., due to non-completion of protocol-specified assessments), or if it was not technically possible to measure body composition parameters from their baseline MRE.

Baseline characteristics, including age, sex, smoking status and history of biological therapy, were collected from Motility study databases. BMI was calculated by dividing weight (in kg) by the square of the patient’s height (in m). BMI was classified as per NHS definition: underweight < 18.5 kg/m^2^, healthy 18.5–24.9, overweight 25–29.9, obese 30–39.9, severely obese ≥ 40 [21].

### Imaging acquisition

Patients underwent MRI at their local hospital according to standard care small bowel MRI protocol (1.5 T or greater). The minimum sequence dataset was: single-shot fast spin echo (SSFSE; HASTE or equivalent), with and without fat suppression, in coronal and axial planes (details of the minimum sequence acquisition can be found in the supplementary material). Intravenous contrast enhanced sequences were permitted but not mandatory.

All patients received oral contrast medium (e.g., mannitol, lactulose) as per local standard care, ingested 40–60 min prior to imaging.

### Imaging analysis

Scans were pseudoanonymised and analysed using Entrolytics (Motilent, London, UK).

**Fig. 1:**
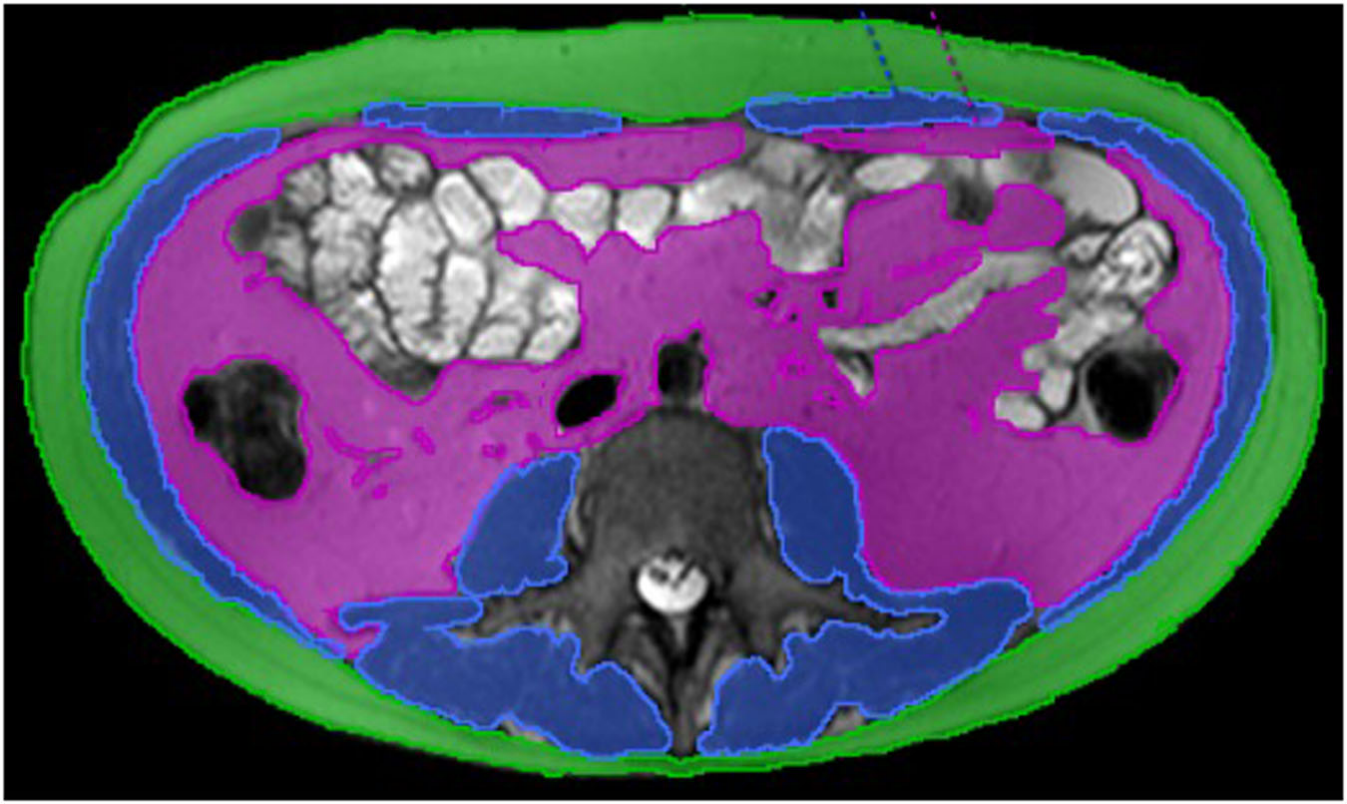
Example of segmentation using a T2 axial image with regions of interest drawn at the L3 vertebral body level, on subcutaneous fat (green), visceral fat (pink), and skeletal muscle (blue)

Using embedded software tools, regions of interest (ROIs) were generated by central-read radiologists (N.S., F.H., and Y.K.) with 7, 5, and 2 years of experience in body MRI, to segment visceral and subcutaneous adipose tissue and skeletal muscle (including abdominal wall, paraspinal and psoas muscles), at the L3 vertebral body level (Fig. 1). ROIs were placed on a single axial T2 acquisition. Readers were blinded to the response group and all clinical information.

The visceral:subcutaneous fat (VAT:SAT) and skeletal muscle:fat (visceral + subcutaneous fat) at L3 were calculated using the derived areas.

Total body fat mass (FM) was estimated according to previously published regression equations [22]:

1 $${Total\; body\; FM}\left({kg}\right)= 	 \, 0.042\times \left[{total\; adipose\; tissue\; at\,L}3\left({{cm}}^{2}\right)\right] \\ 	 +11.2$$

FM was normalised for stature to derive the FM index (kg/m^2^):

2 $${FM\; index}\,({kg}/{m}^{2})=\frac{{Total\; body\; FM}}{{height}\left({m}^{2}\right)}$$

Skeletal muscle (SM) index was normalised for stature to derive the SM index (cm^2^/m^2^):

3 $$L3\,{SM\; index}\,({{cm}}^{2}/{m}^{2})=\frac{{SM\; area\; at\,L}3}{{height}\left({m}^{2}\right)}$$

Myopenia was defined as present in those participants with a sex-specific SM index below the lower quartile of the SM distribution index at baseline. Low skeletal muscle:fat was defined as present in those participants with ratios below the lower quartile of the skeletal muscle:fat distribution at baseline.

### Definition of response or remission

Patients were defined as biologic non-responders on clinical criteria if, after post-induction investigations, they experienced any of: (1) intestinal surgery for the target small bowel disease; (2) necessity to change or stop biological therapy because the treating physician-diagnosed lack of efficacy; or (3) steroid rescue therapy for active luminal CD confirmed by at least one objective test documenting active inflammation (including biochemical, imaging or endoscopic indices).

For the MOTILITY trial, MRE scans at baseline, post-induction and 1 year were interpreted by consultant radiologists with experience of > 100 MRE studies and using MRE routinely. After identifying the most active small bowel segment based on standard morphological features, such as bowel wall thickening and T2 signal, radiologists derived the London Disease Activity Index for each time point for evaluation of RoR. The London Disease Activity Index was calculated as:

1.79 + (1.34 × mural thickness score) + (0.94 × mural T2 signal score) [23].

As part of an exploratory analysis for the MOTILITY trial, the sMaria score was also derived as an alternative MRI activity index to the London Disease Activity index. The sMaria score was calculated as

(1 × thickness > 3 mm) + (1 × oedema) + (1 × fat stranding) + (2 × ulcers) [24].

Patients not fulfilling clinical criteria for nonresponse had RoR defined using ileocolonoscopy or MRE. Specifically, if baseline and 1-year ileocolonoscopy were available, the change in SES-CD (Simple Endoscopic Score for Crohn’s Disease) was used to define RoR. Response was defined as a reduction in SES-CD of ≥ 50% between baseline and 1 year. Remission was defined as a SES-CD score of 0 to 2 [25]. If ileocolonoscopy was not performed at both time points, RoR was defined using the London disease activity index. Response was defined as a ≥ 50% improvement in London Score between baseline and 1 year MRE, and remission was defined as a London score ≤ 4.1 [23]. Analysis was repeated using the sMaria score with response defined as ≥ 50% improvement in sMaria between baseline and 1-year MRE, and remission defined as a sMaria score < 2.

## Statistical analysis

### Study power

The primary power for the MOTILTY study was based on the primary outcome, which was the comparison between the sensitivity of stable or improved mMRI-measured segmental small bowel motility (intervention) vs normalisation of CRP (comparator) at week 12–30 compared to baseline to predict response/remission to biologic therapy at 1 year. The required sample size was 140 patients. The assumed loss to follow-up was 10%, and a recruitment target of 156 patients, but the COVID-19 pandemic prevented many patients from attending protocol-specified procedures. Consequently, interim data monitoring identified loss to follow-up of approximately 30%, necessitating a revised recruitment of 200 patients.

The current study power for this sample size was 95% at alpha = 5% based on logic regression modelling and assuming an odds ratio of over 4.0 for primary nonresponse in the presence of myopenia. A lower OR (2.7), provided 79% power with 140 subjects.

Analyses were conducted according to a prespecified statistical analysis plan and were performed using Stata/MP 18.0 (StataCorp LLC, Texas, USA). Significance was assigned at *p* < 0.05.

The likelihood of response or remission to biological therapy at 1 year was compared between patients with and without skeletal muscle myopenia using a binary logistic regression model. Sensitivity analysis was performed by categorising the patient group according to skeletal muscle:fat (low vs normal), using a similar logistic regression model.

Exploratory analyses assessed the effect of BMI, VAT:SAT, FM index on RoR and sMaria definition of response or remission. All models were adjusted for age at diagnosis, Montreal subtype of disease and presence of perianal disease.

## Results

**Fig. 2:**
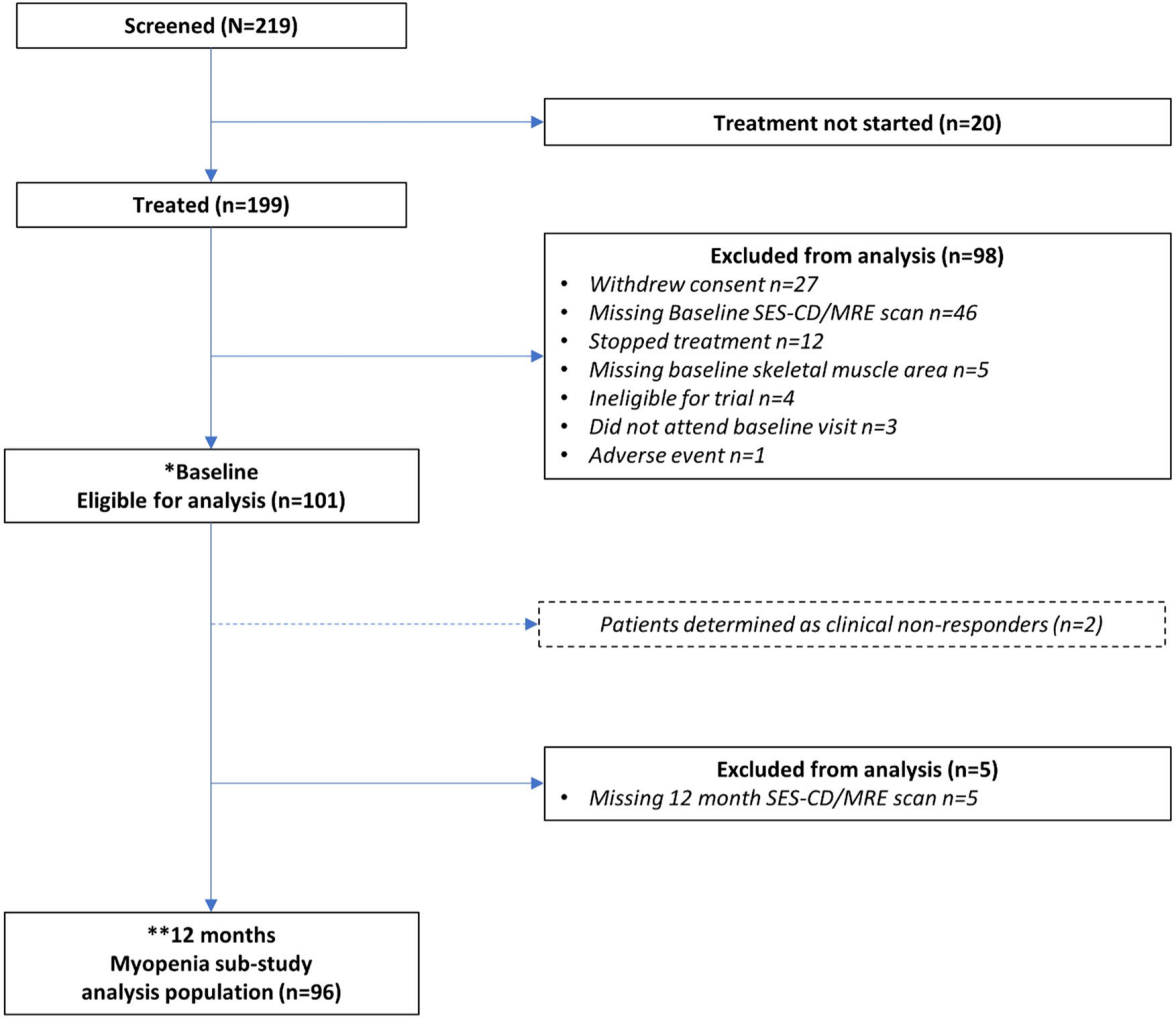
Flow of study participants. * Baseline eligible: includes baseline SES-CD score or baseline MRE score, height, baseline skeletal muscle area, baseline visceral and subcutaneous fat. ** 12-month eligible: includes 12-month SES-CD or MRE score. SES-CD, simple endoscopic score for Crohn’s disease; MRE, magnetic resonance enterography

Ninety-six participants were eligible, following various exclusions that included the negative impact of the COVID-19 pandemic. The flow of study participants is detailed in Fig. 2.

Table 1 describes baseline characteristics of included participants. Average age was 38.2 years (SD 3.8), with 40 (42%) females. Most participants, 65%, had no previous bowel surgery and 82% had no prior biological therapy.

**Table 1:** Baseline characteristics of the 96 study participants

Baseline characteristics	Mean	(sd)
Age (years)	38.2	(3.8)
	*n*	(%)
Gender		
Female	40	(41.67)
Male	56	(58.33)
Body mass index (BMI)		
Underweight (< 18.5)	6	(6.25)
Healthy range (18.5–24.9)	51	(53.12)
Overweight (25–29.9)	22	(22.92)
Obesity/severe obesity (> 30)	10	(10.42)
Missing	7	(7.29)
Smoking status		
Non-smoker	45	(46.88)
Current smoker	12	(12.50)
Ex-smoker	15	(15.62)
Missing	24	(25.00)
Previous bowel surgery		
No surgery	62	(64.58)
Single surgery	19	(19.79)
Multiple surgeries	15	(15.62)
History of biological therapy		
No	79	(82.29)
Yes	17	(17.71)
Type of biologic		
Anti-TNF	74	(77)
IL inhibitor	17	(18)
Other/missing	5	(5)
Age at diagnosis (years)		
A1 (≤ 16)	10	(10.42)
A2 (17–40)	69	(71.88)
A3 (> 40)	17	(17.71)
Location		
L1 (ileal)	63	(65.62)
L3 (ileocolonic)	33	(34.38)
Behaviour		
B1 (non-stricturing, non-penetration)	51	(53.12)
B2 (stricturing)	32	(33.33)
B3 (penetrating)	12	(12.5)
Missing	1	(1.04)
Perianal disease modifier (p)		
No	87	(90.62)
Yes	8	(8.33)
Missing	1	(1.04)
SES-CD score	6.0	(4.9)
MRE activity score (London activity index)	6.3	(1.7)
Skeletal muscle area (cm^2^)	68.8	(22.2)
Skeletal muscle index (cm^2^/m^2)^	23.4	(7.4)
VAT:SAT	0.5	(0.4)
Total fat area (cm^2^)	108.4	(67.3)
Fat mass index (kg/m^2^)	5.4	(1.2)

*SES-CD* simple endoscopic score for Crohn’s disease, *MRE* magnetic resonance enterography, *VAT:SAT* visceral:subcutaneous fat

### Prognostic value of skeletal muscle index and skeletal muscle:fat at baseline for RoR at 1 year

Thirty-four (35%) patients were deemed responders at 1 year; two based on clinical criteria and the remainder on MRI activity scores (London Activity index). Sex-specific cut-points based on the lower quartile (25%) of skeletal muscle area at baseline in this cohort were used to define myopenia. We used 61.05 for males and 44.34 for females. The cut-point for low skeletal muscle:fat based on the lower quartile at baseline was 0.4091. Complete case analyses based on 94 participants with available data on confounding factors at baseline are presented in Table 2. The low skeletal muscle:fat analysis is based on 72 patients with available total fat data.

**Table 2:** Difference in RoR (using London activity index definitions) to biological therapy at 1 year for patients with and without skeletal muscle myopenia and patients with and without low skeletal muscle:fat

	*N*	Response or remission *N* (%)	Odds ratio (95% CI)*	*p*-value
Skeletal muscle myopenia				
Without	69	25 (36.2)	Reference	-
With	25	7 (28.0)	0.85 (0.27–2.66)	0.78
Skeletal muscle:fat				
Normal	53	17 (32.1)	Reference	-
Low	19	4 (21.1)	0.71 (0.19–2.71)	0.62

Note that some participants were not included in the low skeletal muscle:fat analysis as total fat data were missing

* Model adjusted for age at diagnosis, Montreal subtype of disease and presence of perianal disease

The likelihood of having RoR at 1 year (using the London Activity index definitions) was not significantly different between patients with and without skeletal muscle myopenia and those with or without low skeletal muscle:fat (Table 2).

### Exploratory analyses

The likelihood of having RoR at 1 year (using the sMaria index definitions) was not significantly different between patients with and without skeletal muscle myopenia, and those with or without low skeletal muscle:fat (Table 3).

**Table 3:** Difference in RoR (using sMARIA definitions) to biological therapy at 1 year for patients with and without skeletal muscle myopenia and patients with and without low skeletal muscle:total fat ratios

	*N*	Response or remission *N* (%)	Odds ratio (95% CI)*	*p*-value
Skeletal muscle myopenia				
Without	71	28 (39.4)	Reference	-
With	25	10 (40.0)	1.70 (0.59–4.87)	0.33
Skeletal muscle:total fat				
Normal	55	24 (43.6)	Reference	-
Low	19	3 (15.8)	0.27 (0.07–1.13)	0.07

Note that some participants were not included in the low skeletal muscle:total fat analysis as total fat data were missing

* Model adjusted for age at diagnosis, Montreal subtype of disease and presence of perianal disease

There was no significant difference between the likelihood of RoR in different BMI categories when compared with the healthy range. There was no association between FM index or VAT:SAT and RoR (Table 4).

**Table 4:** Difference in RoR to biological therapy at 1 year by BMI category (compared to the healthy range), fat mass index and VAT:SAT

Baseline factor	*N*	Response or remission *N* (%)	Odds ratio (95% CI)*	*p*-value
Body mass index (BMI)				
Healthy range (18.5–24.9)	50	18 (36.0)	Reference	-
Underweight (< 18.5)	6	2 (33.3)	1.08 (0.15–7.59)	0.94
Overweight (25–29.9)	21	7 (33.3)	0.85 (0.28–2.63)	0.78
Obesity/severe obesity (> 30)	10	2 (20.0)	0.44 (0.07–2.55)	0.36
Fat mass (FM) index	67	48 (71.6)	0.68 (0.40–1.15)	0.15
Visceral-to-subcutaneous fat ratio (VAT:SAT)	72	51 (70.8)	1.90 (0.52–6.91)	0.33

Note that some participants were not included in the analyses as a result of missing data (fat area on MRI or height or weight for BMI calculation)

* Model adjusted for age at diagnosis, Montreal subtype of disease and presence of perianal disease

## Discussion

In this multicentre, prospective cohort study of 96 patients with active SBCD, body composition parameters (specifically myopenia, skeletal muscle:fat, VAT:SAT and FM index) measured using MRI did not predict biologic therapeutic RoR at 1 year. Additionally, the clinical measure of body composition, BMI, was also unable to reliably predict RoR at 1 year. Overall, in the adjusted model, the odds of reaching RoR were 15% lower in the patients with myopenia compared to those without, not statistically significant.

Multiple studies have investigated body composition parameters, predominantly skeletal muscle mass and visceral adiposity, in patients with CD. Most have focussed on disease course or clinical outcome (such as severity or behaviour, e.g., stenosis or penetration, need for surgery, or complications after surgery). A few studies have investigated body composition changes after treatment; patients with myopenia may increase muscle mass after biologics [26–28], and after infliximab induction, the visceral adiposity in patients with mucosal healing reduces significantly [29].

Only a handful of studies have investigated body composition as a predictor of RoR to biologics. In a study of 106 patients with CD, Ding et al [19] found that myopenia (but not visceral adiposity) was associated with primary nonresponse to anti-TNF therapy. This finding contrasts with ours, which found no association between myopenia and RoR. The two studies differed in their design (Ding et al was retrospective and single site whereas ours was prospective and multisite), and in how RoR was defined (Ding et al used a global physician assessment to define primary nonresponse). To our knowledge, ours is the first study to include MRI activity score definitions for response or remission. Such scores are validated, and we analysed the data using both the London activity index and the sMaria score. However, optimal definitions of response and remission for such scores remain under investigation. Additionally, Ding et al did not account for potential confounders such as age, perianal disease, and Montreal disease classification and did not report a power calculation for the study. A further single-centre, retrospective study found that time to treatment failure was significantly faster in patients with CD with myopenia compared to those without [20]. Again, the study design differed from ours (for example, in using MRI or CT scans) and potential confounders were not accounted for. Another reason potential for our negative findings is the nature of the patients recruited to the Motility trial. Overall, 33% had a stricturing phenotype, and medical therapy is not effective against fibrotic disease. We did attempt to mitigate this by excluding patients with upstream small bowel dilation who are more likely to have fibrotic stricturing. There is also good data showing biologics are effective in those with inflammatory strictures [30].

A hypothesis for treatment failure in patients with myopenia and visceral adiposity is diminished drug distribution; it would be interesting to measure drug levels in future studies. One study has investigated drug levels in CD patients related to visceral adiposity; Lim et al found that increased visceral fat is associated with lower infliximab trough concentrations [31], implying that individuals with visceral obesity may require higher doses to achieve therapeutic levels. A further study found that VAT volume is associated with anti-TNF alpha response in a non-dose-dependent manner [32]. Another hypothesis is that fat is pro-inflammatory with adipocytes in the mesenteric fat producing c-reactive protein (CRP) in response to local inflammation in CD [33], so that patients with more visceral fat have a greater inflammatory response.

As a prespecified substudy of the MOTILITY trial, the strengths of the current study are its prospective, multicentre design, and use of prespecified thresholds for RoR. As noted above limitations include using a MRE-based definition of RoR (rather than SES-CD via ileocolonoscopy). A minority of participants were not biologic treatment naïve, and patients with prior exposure to biologics and/or on additional immunomodulators may have different responses. We did not collate the ethnicity of the recruited patients, and it would have been useful to analyse the data using different body composition criteria applicable to different ethnic groups. We attempted to mitigate this by using the spread of the data in the recruited cohort to define the lower quartile of body composition metric rather than using pre-defined cutoffs from the literature which may not be applicable to our patient cohort. Although the sample size was reasonable, the study was significantly impacted by the COVID-19 pandemic, which resulted in a smaller number of participants recruited than originally planned and a higher loss to follow-up. At 96, our sample size was lower than the 140 anticipated, which impacted our ability to detect smaller predictive effects. However, our data gave no clear suggestion of any predictive ability of body composition parameters. We also were not able to explore potential differences in anti-TNFα and anti-IL-12/23 therapy classes due to a lack of statistical power. Whilst 2-dimensional analysis of body composition at the level of the L3 vertebral body has been validated in previous studies and has high inter-reader agreement [22, 34], 3-dimensional volumetric segmentation of whole-body fat and muscle has the potential to increase accuracy. This is possible with whole-body MRI scans (with reduced acquisition times) and dedicated post-processing software; acquisition of a whole-body MRI scan could be incorporated into future studies. Overall, larger multicentre studies that address the various potential cofounders are needed to fully investigate the link between body composition and response to therapy in Crohn’s disease.

In conclusion, we found that body composition parameters at baseline were unable to predict RoR at 1 year.

## Supplementary information

ELECTRONIC SUPPLEMENTARY MATERIAL

## Data Availability

Requests for data will be considered by the Chief Investigators.

## Abbreviations

anti-TNFα: Anti-tumour necrosis factor alpha
BMI: Body mass index
CD: Crohn’s disease
CT: Computed tomography
FM: Fat mass
IBD: Inflammatory bowel disease
mMRI: Motility MRI
MRE: Magnetic resonance enterography
NHS: National Health Service
ROI: Region of interest
RoR: Response or remission
SBCD: Small bowel Crohn’s disease
SES-CD: Simple endoscopic score for Crohn’s disease
SM: Skeletal muscle
VAT:SAT: Visceral:subcutaneous fat
