# Supplementary material for: MRI assessment of body composition for prediction of therapeutic response to biologic agents in patients with Crohn’s disease
Source: Insights Imaging. 2025 Mar 19;16:61. doi: 10.1186/s13244-025-01930-w (PMC11923306; doi:10.1186/s13244-025-01930-w)
Supplement: Supplementary file 1 — ELECTRONIC SUPPLEMENTARY MATERIAL [file 13244_2025_1930_MOESM1_ESM.pdf]

MRI assessment of body composition for prediction of therapeutic response to biologic agents in patients with Crohn’s disease
ELECTRONIC SUPPLEMENTARY MATERIAL

Supplementary material 1.
Minimum set of anatomical sequences to be acquired at each time point.

Imaging type	Anatomical	Anatomical	Anatomical
Sequence	T2 - Coronal	T2 - Axial	T2 – Coronal OR Axial
Fat Suppression	No	No	Yes
Example	HASTE	HASTE	FS HASTE

Abbreviations: HASTE Half-Fourier Acquisition Single-shot Turbo spin Echo; FS fat suppression
